# Nanomaterials in the Context of Type 2 Immune Responses—Fears and Potentials

**DOI:** 10.3389/fimmu.2017.00471

**Published:** 2017-04-25

**Authors:** Martin Himly, Robert Mills-Goodlet, Mark Geppert, Albert Duschl

**Affiliations:** ^1^Division of Allergy and Immunology, Department of Molecular Biology, University of Salzburg, Salzburg, Austria

**Keywords:** allergy, immunomodulation, immunotherapy, nanomedicine, nanoparticles, parasite infection, vaccine, wound healing

## Abstract

The type 2 immune response is an adaptive immune program involved in defense against parasites, detoxification, and wound healing, but is predominantly known for its pathophysiological effects, manifesting as allergic disease. Engineered nanoparticles (NPs) are non-self entities that, to our knowledge, do not stimulate detrimental type 2 responses directly, but have the potential to modulate ongoing reactions in various ways, including the delivery of substances aiming at providing a therapeutic benefit. We review, here, the state of knowledge concerning the interaction of NPs with type 2 immune responses and highlight their potential as a multifunctional platform for therapeutic intervention.

## Nanomaterials and Type 2 Immune Responses

Upon contact with non-self entities, the adaptive immune system decides between one of three response programs. The tolerance program, orchestrated by regulatory T cells (T_reg_), ensures that no defense is initiated against harmless agents. If pathogens are identified, the adaptive immunity chooses between two main types of defensive responses (1). The first branch, a type 1 response, is characterized by the rapid removal of pathogens by macrophages and neutrophils, mediated by T helper 1 (T_H_1) and T_H_17 cells, which release pro-inflammatory cytokines, such as interferon (IFN)-γ and interleukin (IL)-12. Type 1 responses are integrated seamlessly with inflammatory reactions. The role of inflammation and type 1 responses in the context of exposure to nanoparticles (NPs) is discussed elsewhere in this volume.

The second defensive branch, type 2 immunity, involves the key cytokines IL-4, IL-5, IL-13, and different types of immune cells, such as basophils, eosinophils, mast cells, anti-inflammatory (M2) macrophages, and T_H_2 cells (1). This type of response is often connected to parasitic infections, later stages of the wound healing process, and to chronic inflammatory conditions, such as asthma and allergy (2). Of note, some NPs are known to modulate type 2 immune responses (3). This review covers applications of NPs in the context of type 2 immune responses, such as parasitic infections, wound healing, and allergy, with a special focus on therapeutic approaches.

## Parasitic Infections

Ancestral populations can be assumed to have been constantly subjected to parasite infections. Hence, macroparasites have played a large role in the evolution of type 2 immune responses. One particular purpose of type 2 responses is to limit the parasite load and is done so, *via* immunoglobulin (Ig)E type antibodies and effector cells (4). Parasitic diseases continue to be a serious health problem in large areas of the world (5). Unfortunately, there are currently no studies regarding coexposure to parasites and nanomaterials. However, nanomedical approaches have been investigated for vaccination, diagnosis, and therapy of parasitic diseases (6–8). Some studies have looked specifically at a shift between type 1 and type 2 responses, as indicated by characteristic cytokines and antibody isotypes. In particular, numerous nanomedical studies concerning malaria have been performed, including studies about the response type (7). For example, self-assembled protein NPs were used to vaccinate mice with *Plasmodium sp*. antigens, resulting in the development of protective type 2 responses (9).

In contrast, chondroitin nanocapsules upregulate T_H_1 cytokines and downregulate T_H_2 cytokines in hamsters, leading to enhanced doxorubicin-induced apoptosis that eradicates infection with *Leishmania donovani* (10). Similarly, the host response of mice against *L. donovani* was supported by artemisinin-loaded NPs that shifted the cytokine profile from type 2 to type 1 (11). This corresponds to the conventional view that *Leishmania*, like other microparasites, is promoted by type 2 responses and controlled by type 1 responses. However, it should be borne in mind that careful analysis of this mouse model has revealed that the prototypic T_H_2 cytokines IL-4 and IL-13 can contribute to either the control or the exacerbation of disease (12). It is, thus, not always clear which role type 2 responses play in relation to specific parasites. NP adjuvants contribute to effective vaccination of mice against *Angiostrongylus costaricensis* and of pigs against *Trichinella spiralis*, but they do this by supporting a type 1 response in the first case and a type 2 response in the second (13, 14). Altogether, it is clear that NPs can influence the type of immune response toward a challenge, with either detrimental or protective effects for the host.

## Wound Healing

Wound healing is a natural process that repairs and regenerates damaged tissues, for example, in the skin (15), lung (16), or intestine (17). Numerous therapies have been developed to accelerate this process, involving, for example, pharmaceutics, stem cells, electrical stimulation, negative pressure, light, or radiation (15, 18–21). Furthermore, NPs, especially those with antimicrobial properties, are considered as valuable tools in accelerating the wound-healing process (22). Silver (Ag) was used for its antibacterial properties since the Roman empire, and nowadays, numerous therapeutical products containing ionic Ag or Ag NPs are on the market (22, 23). Several publications review the beneficial effects of ionic nanoparticulate Ag in wound healing (22, 24, 25). An earlier animal study by Tian et al. (26) showed that Ag NPs accelerate healing and improve cosmetic appearance of wounds in a dose-dependent manner. By analyzing bacterial growth and cytokine profiles in wound sections, the authors demonstrated the antimicrobial and anti-inflammatory potential of Ag NPs. Microbially synthesized Ag NPs enhanced wound-healing efficiacy in rats (11, 27, 28). Using a transforming growth factor (TGF)-β receptor inhibitor, Li and coworkers proposed the activation of the TGF-β1/Smad signaling pathway as a mechanism of wound-healing enhancement by polyvinylalcohol/chitosan oligosaccharide Ag nanofibers (29).

Gold (Au) NPs were successful in acceleration of wound healing in combination with photobiomodulation therapy in rats (15) or in combination with the antioxidants epigallocatechin gallate (EGCG) and α-lipoic acid (ALA) in mice (30). The observed decrease of CD68 expression and increase of SOD1 expression around the wound area suggest that anti-inflammatory as well as antioxidative effects of the Au NP/EGCG/ALA mixture play a role in increased wound-healing efficiency (30). The inflammatory reaction in wounded skin of rats was investigated in a recent report. Phytochemically stabilized Au NPs accelerate wound healing in a process that involves alteration of the amounts of TGF-β1, vascular endothelial growth factor (VEGF), and the number of mast cells in the wounded skin sections compared to vehicle controls (31, 32). These observations indicate an involvement of the particles in type 2 immune functions during the healing process. A different approach for wound healing with Au NPs in diabetic mice, showed that spherical nucleic acid–Au NP conjugates efficiently to downregulate target genes in diabetic mice. Thus, resulting in full wound closure occurring within 12 days, compared to control wounds which were only 50% closed (33).

Aside from Ag and Au, other types of NPs, such as selenium (34), zinc oxide (35), copper oxide (36, 37), iron oxide (38), or polymeric NPs (39), were shown to be beneficial for wound healing (Table 1). Thereby, the beneficial effect is either a result of the NPs properties alone (i.e., antibacterial effects) or a combined result of the NPs with other substances. For example, TiO_2_ NPs have been shown to enhance the wound-healing potential of chitosan (40), which is used as wound dressing material (41) and is currently commercially available (42). Some caution may be necessary when using very high concentrations of chitosan leading to a highly positively charged NP surface, as recently demonstrated in a study involving Au NPs (43). Increased uptake by phagocytic cells and an enhanced pro-inflammatory response were determined, rendering chitosan coating exceeding an optimal range counteractive for wound healing. Chitosan-based copper nanocomposites accelerate wound healing in rats by modulation of different cytokines and growth factors. The upregulation of VEGF, TGF-β1, and IL-10 as well as the downregulation of tumor necrosis factor α (TNF-α) indicate a shift toward type 2 immunity. An interesting approach using biodegradable NPs was published by Galili (44), who demonstrated that α-Gal NPs can accelerate the process of wound healing. The mechanism involves binding of natural anti-α-Gal antibodies to the multiple α-Gal epitopes, which then present on the NPs resulting in complement activation, recruitment, and activation of macrophages, which leads to tissue regeneration (44, 45). A summary of current therapeutic approaches for NPs is given in Table 1.

**Table 1:** **Selected therapeutic nanoparticle (NP)-based approaches in the context of type 2 immune responses at different stages of development**.

Nanomaterial type	Therapeutic benefits	Reference
**In clinical practice**		
**Inorganic NPs**		
Silver	Most widely used NPs in wound healing due to their antimicrobial and anti-inflammatory properties. Several products already on the market	(22–24, 26)
**Organic/biodegradable NPs**		
Glatiramer acetate	Prolonged onset and reduced transition from relapsing remitting to progressive multiple sclerosis	(46, 47)
Lipids	T cell inhibition and immunosuppression by encapsulating sirolimus into nanostructured lipid carriers	(48)
**In clinical studies**		
**Organic/biodegradable NPs**		
l-leucin-l-glutamate copolymers	Enhanced depot effect for insulin upon subcutaneous injection	(49)
Polyethylene glycol (PEG)	Anti-tumor necrosis factor α antibody fragment against rheumatoid arthritis and Crohn’s disease	(50)
Calcium phosphate	Enhanced depot effects for various drugs	(51)
Poly-l-lysine dendrimer	Antimicrobial protection from genital herpes and HIV infection	(52)
Virus-like particles (VLPs)	VLPs derived from Qbeta bacteriophages filled with CpG-DNA and filled with house dust mite extract, respectively, conjugated with Der p 1 peptide	(53, 54)
**In development/basic research studies**		
**Inorganic NPs**		
Gold	Successful acceleration of wound healing in combination with photobiomodulation therapy, antioxidants, or nucleic acids Phytochemically stabilized Au NPs accelerate wound healing altering the amounts of transforming growth factor *Plasmodium falciparum* antigen Pfs25 or *Yersinia pestis* F1	(15, 30–33, 55, 56)
Cerium oxide	Acceleration of the wound-healing process by enhancement of the proliferation and migration of fibroblasts, keratinocytes, and vascular endothelial cells	(57)
Selenium	Shortening of healing duration of artificial wounds in Wistar rats	(34)
Zinc oxide	Castor oil/chitosan-modified ZnO NPs increase wound-healing efficacy in rats	(35)
Copper oxide	Enhanced wound-healing activity of CuO NPs by inhibiting pathogenic bacteria surviving in the wound sites	(36, 37)
	Acceleration of wound healing by chitosan-based copper nanocomposites involves a type 2 shift of immune response	
Iron oxide	Thrombin-conjugated magnetic γ-Fe_2_O_3_ NPs enhance wound healing in rats	(38, 58)
	Reeducation of TAMs from M2 toward M1 phenotype by FDA-approved ferumoxytol	
Titanium dioxide	TiO_2_ NPs enhance wound-healing potential of chitosan	(40)
Fullerene	Induction of dendritic cells (DCs) maturation and activation of T_H_1 immune response using [Gd@C_82_(OH)_22_]*_n_* fullerene NPs	(59)
Silica	Boost of vaccine immune response against influenza virus	(60, 61)
	Lysozyme-loaded mesoporous silica NPs (nanopollens) with long-term antibacterial effects tested in *ex vivo* small intestine models	
Carbon nanotubes (CNTs)	*Plasmodium vivax* AMA-1 N-terminus peptide–CNT conjugate delayed parasitemia in infected *Plasmodium berghei* mouse model	(62)
**Organic/biodegradable NPs**		
Chondroitin	Doxorubicin-loaded chrondroitin nanocapsules eradicate infection with *Leishmania donovani* in hamsters	(10)
Polyglutamic acid (PGA)	Timothy grass pollen extract-loaded PGA NPs as delivery vehicle to DCs	(63)
Poly-d,l-lactic-co-glycolic acid (PLGA)	Inhibition of T_H_2 immune response and airway inflammation in mice	(11, 64–71, 72)
	Treatment for autoimmune disease by induction of antigen-specific tolerance using myelin bound to NPs	
	Reprogramming of TAMs by rabies virus glycoprotein peptide-loaded paclitaxel-carrying NPs in a mouse glioma model	
	CpG/peanut extract-PLGA enhance peanut-specific immunotherapy	
	Bet v 1-loaded PLGA NPs improve efficacy of allergen-specific immunotherapy (AIT) by downregulating ongoing T_H_2 response in mouse models	
	Ole e 1-loaded PLGA (<2 μm) microparticles as vehicle for AIT	
	Oral administration of major *Chenopodium album* pollen allergen Che a 3-PLGA downregulates T_H_2 response in mouse model	
	Artemisinin-loaded PLGA NPs showed superior antileishmanial efficacy compared to free artemisinin in a mouse model and shifted cytokine profile from type 2 to type 1	
	Successful M cell targeting with birch pollen allergen-loaded PLGA NPs specifically functionalized with *Aleuria aurantia* lectin	
Polymethylvinyl ether-co-maleic anhydride (PVM-MA)	Ryegrass pollen extract-loaded PVM-MA NPs as adjuvant for AIT	(73)
PEG	Self-assembled PEG-dendrimer efficiently delivered and increase anti-inflamatory effect of dexamethasone in allergic airways inflammation pH-sensitive PEG nanocarriers for grass pollen and house dust mite allergen encapsulation and controlled release from DCs	(74, 75)
Chitosan	Local nasal AIT with house dust mite-chitosan vaccine in mouse asthma model Intranasal AIT with immunodominant Der p 1 epitope reduced allergen-specific T cell reactivity and interleukin (IL)4 and IL5 levels in brochnoalveolar fluid of sensitized mice Oral DNA vaccine of house dust mite allergen Der p 1 formulated with chitosan NPs Induction of T_H_1 immune response by DNA vaccine of Der p 2 with chitosan NPs Oral gene delivery of chitosan-formulated NPs in peanut-allergic mouse model with additional induction of mucosal dimeric allergen-specific immunoglobulin A	(76–80)
Polyanhydride NPs	Intradermal immunization of mice with polyanhydride NPs loaded with peanut proteins induced strong mixed T_H_1/T_H_2 immune response (immunostimulant)	(81)
Polyacrylic acid	Antibacterial activity of poly-phospoester-based Ag-loaded NPs in lung infections	(82)
Protamine NPs	Liposome–protamine–DNA NPs induced strong T_H_1 response upon subcutaneous AIT in *Chenopodium album*-sensitized mouse model	(83, 84)
	Protamine-based NPs (proticles) with CpG complexed with Ara h 2 extracted from raw peanuts induced strong T_H_1 response upon subcutaneous AIT in mice	
Self-assembled protein NPs (SAPN)	SAPN used to vaccinate mice with *Plasmodium sp*. antigens achieved delayed parasite motility and complement lysis	(9)
Immunostimulatory complexes (ISCOMs)	Effective intranasal immunization of mice against *Angiostrongylus costaricensis* with ISCOM formed by a synthetic pph 1 peptide linked to cholera toxin adjuvanted with saponin/phospholipids/cholesterol NPs	(14)
α-Gal NPs	Tissue regeneration induced by macrophages activated through binding of natural anti-α-Gal antibodies to multiple α-Gal epitopes present on the NPs	(44, 45)

## Allergy

Allergy and asthma represent a global public health concern in developed countries, with a steady increase also occuring in emerging countries. According to the World Health Organization, approximately 300 million people worldwide are currently suffering from asthma, with a rising trend to increase up to 400 million by 2025 (85). Allergic diseases include the various forms of asthma, rhinitis, conjunctivitis, angioedema, urticaria, eczema, eosinophilic disorders, such as esophagitis and life-threatening anaphylaxis, as in the case of food, insect venom, or drug allergies. Patients with allergic diseases have a significantly reduced quality of life, and even milder forms such as allergic rhinitis have a significant economic impact (86). Globally, allergic diseases affect 20–30% of the population, and in the developed countries sensitization rates of up to 50% have been reported (87).

Allergy is defined by IgE reacting specifically with non-pathogenic environmental proteins, thus, being defined as allergens (88). Presence of allergen-specific IgE in the blood of affected individuals resulting from an overshooting T_H_2-driven immune response, is hence the hallmark of being sensitized (89). The sensitization process is intiated upon first contact where a variety of potential functions of allergens may be involved (90–98); however, the overall mechanism of allergic sensitization still remains to be fully established. As potential risk factors, nutrition, and hygiene have been described (99). Upon second contact with the allergen, specific IgE-loaded allergic effector cells, i.e., tissue-resident mast cells and peripheral blood basophils, degranulate due to IgE receptor cross-linking and release vasoactive mediators (histamine, tryptase, etc.). During this process, being termed the effector function, the typical allergic symptoms emerge, including vasodilation and permeation resulting in swelling, itching, and redness, characteristic of the *wheal and flare reaction* in rhinoconjunctivitis. Furthermore, effector cells initiate the secretion of lipid mediators (leukotrienes) and cyto-/chemokines leading to bronchoconstriction, mucus production, intestinal hypermotility, as in the case of more severe forms, such as anaphylaxis (88). Furthermore, eosinophil infiltration, chronicity, and amplification of the allergic response can lead to tissue remodeling, a characteristic of asthma (100).

Presently, few studies investigating the potential sensitization-aggravating effects of particulate matter itself or NP-associated allergens exist (101–103). Historically, research was conducted on combustion-derived particles as reviewed recently (104, 105). The interaction of allergens with engineered NPs, such as Au, Ag, ZnO, TiO_2_, SiO_2_, may arise at sites where such materials are handled, so risk of disease-aggravating conditions can be expected in occupational settings. Studies in mice have addressed the pro-allergic potential of Au, TiO_2_, and SiO_2_ NPs in contact hypersensitivity. Such reactions are characterized by a T cell-mediated delayed-type adverse response without the presence of allergen-specific IgE or airway hyperresponsiveness with eosinophil infiltration, mucous cell metaplasia, and elevated type 2 cytokine secretion (106–108). Graphene nanosheets and multiwalled carbon nanotubes (MWCNTs) have been shown to induce a T_H_2 immune response in mouse models when administered intravenously (109). While in human *in vitro* studies including fullerene or MWCNTs contrasting results were reported (110, 111). Human skin-derived mast cells and peripheral blood basophils exhibited a significant inhibition of IgE-dependent mediator release by fullerene. Furthermore, MWCNTs were shown to inhibit allergen-induced type 2 cytokine secretion by peripheral blood mononuclear cells from house dust mite-allergic individuals, emphasizing the pro-inflammatory potential of MWCNTs which has recently been reviewed (112). In line with these reports, MWCNTs have been shown to suppress humoral immune effects in mice by a mechanism involving the activation of cyclooxygenases in the spleen in response to signals from lung (113). Accordingly, iron oxide NPs were shown to attenuate serum levels of OVA-specific IgG_1_ and IgG_2a_ in mice (114). Protein corona formation represents a paradigm when studying the biological effects of NPs and it is well accepted that protein–NP interactions may alter the proteins’ 3D structure and hence epitope integrity (115). In the context of type 2 immune effects, IgE epitope integrity is essential. Following this rationale, allergic disease-modulating effects were investigated upon interaction of three major inhalant allergens with Au NPs (116). This study showed that increased, decreased, or similar allergenic responses may be observed, presumably depending on the orientation and accessibility of the IgE epitopes of the allergens bound to the NPs.

Not only material composition has an influence on the type of immune response but the particle size of the same material can also be decisive upon inducing either a type 1 or a type 2 immune response. Bigger particles (>100 nm) are more prone to induce a type 2 response, in comparison to smaller particles (~50 nm) that rather induce a type 1 response (117, 118). Wen et al. showed that NPs were also able to induce both a T_H_1 and a T_H_2 response equally when using chitosan NPs in combination with ovalbumin in mice (119). The immune responses elicited by different NPs can be diverse and are highly dependent on material and size of the particles.

During the past two decades much progress has been made in the field of molecule-based diagnostics, also termed component-resolved diagnostics (CRD), with the development of two types of serological tests involving purified natural or recombinantly produced allergen molecules, coated to particles (ImmunoCAP™) or a glass surface (ISAC™) (120–122). The higher predictive value of CRD compared to extract-based methods has been appreciated by clinicians (123, 124). These two large studies advocate that CRD improves the decision-making process during the prescription of allergen-specific immunotherapy (AIT) due to its high specificity. AIT has been described >100 years ago and still remains the only effective treatment against allergy resulting in a shift from a type 2 immune response toward a tolerogenic state, which is characterized by the key cytokines IFN-γ, IL-10, and TGF-β and production of allergen-specific IgG_4_ blocking antibodies (125–127). The potential of NPs being used for allergen therapeutics emerged from adjuvants which will be discussed next.

## Adjuvants

The idea to use adjuvants to aid in vaccination was established due to the finding that a higher specific antibody titer can be induced by an abscess at the site of inoculation (128, 129). Adjuvants comprise different classes of compounds, including microbial substances, mineral salts, emulsions, or microparticles, displaying potentiating and/or modulating effects on the human immune system, and they have even been quoted as *“dirty little secrets of immunologists”* (130, 131). The main desired effects of adjuvants in therapy or vaccination can be broken down into two groups. On the one hand they function as delivery vehicles of the active pharmaceutical ingredient (API) to antigen-presenting cells (APCs), like dendritic cells (DCs) and macrophages. On the other hand, they induce an immune potentiation effect that is achieved by activation of the APCs through specific receptors, thus creating an inflammatory context (132). Adjuvants have to be safe in formulation, stable during storage, easily expelled from the body, either by being biodegradable or by efficient excretion, and furthermore, the costs of their production should to be low (133).

Aluminum hydroxide or alum has been in use as an adjuvant from as early as 1926 (134), widely used in vaccination ever since (135). Its clinical function also involves innate mechanisms established for recognition of crystals based on NLRP3 inflammasome activation (136). In the last two decades, the research into new adjuvants has increased, but many new adjuvants fall prey to local or systemic toxicity and are not suitable for the use in humans (137). A possible new approach is the use of nanosized inorganic or organic particles as an efficient antigen delivery vehicle (138, 139). Additional advantages of using NPs as adjuvants are that they can incorporate several desired effects of an adjuvant in one substance. They may (i) confer a depot function with enhanced abundance in the tissue/circulation, (ii) function as a delivery vehicle by binding the APIs and delivering them to the APCs, and (iii) be able to induce immunostimulatory effects (140). It has been demonstrated that different kinds of NPs ranging from inorganic NPs, like silica (60, 141) and gold (142), over lipids (143) to biodegradable polymeric particles (144, 145) show adjuvant potential. For some NPs the adjuvant effect is greater than that of alum (138, 141, 146).

Due to their unique properties, NPs readily bind substances like proteins, peptides, and nucleic acid vaccines (147). Those conjugates have been shown to be taken up by APCs (146, 148), and thus NPs are able to deliver the APIs to the APCs. The immune stimulatory effect of NPs has been shown, for example, using poly-γ-glutamic acid NPs and DCs (148), which facilitates the second major requirement for adjuvants—to provide a costimulatory signal for initiation of an immune response. Keeping all that in mind, several types of NPs bear the potential to act as efficient adjuvants in formulation.

## NPs—A Potential Multifunctional Platform for Interactions with the Immune System

**Figure 1:**
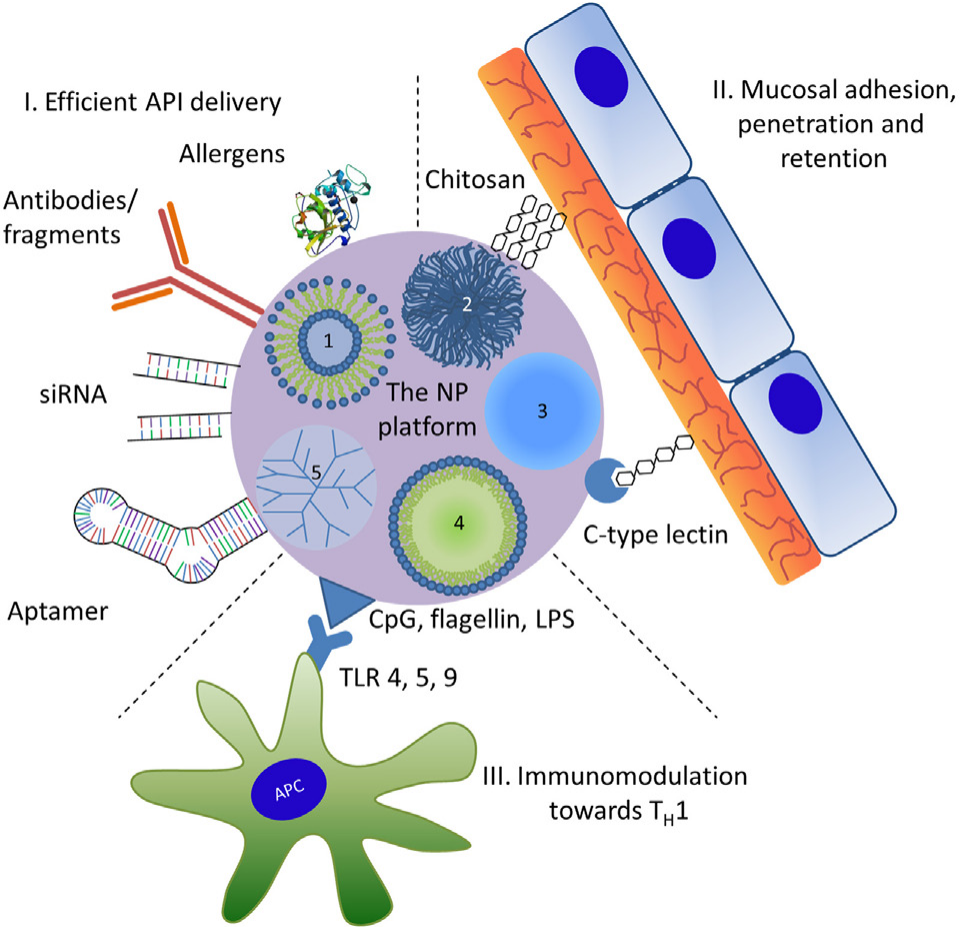
**Nanoparticles (NPs) used as a potential multifunctional nanomedical platform to facilitate three roles in therapeutic use**. The numbers represent different types of NPs: 1, liposomes; 2, biopolymers; 3, inorganic NPs; 4, nanoemulsions; 5, dendrimers. Active pharmaceutical ingredient (API); for symbolizing protein APIs the 3D structure of Der p 1, the major house dust mite allergen (PDB entry 3f5v) was used; LPS, lipopolysaccharide; TLR, Toll-like receptor; T_H_1, T helper 1 cells.

In addition to spontaneous interactions of proteins (or other biological substances) with NPs, engineered nanomaterials may form a platform where various functions of different chemical entities may be combined intentionally (Figure 1). It should be stated here that in particular for nanomedical approaches the strict nano definition by “ISO/TS27687:2008 *Nanotechnologies—Terminology and definitions for nanoobjects—NP, nanofibre and nanoplate”* confining NPs for a size range up to 100 nm is often relaxed. Therefore, nanomedicines usually list substances of particulate matter in the submicro size range. The surface of NPs can be functionalized covalently with specific ligands including antibodies and fragments thereof or other immunologically active proteins, such as allergens. Other ligands may include peptides, nucleic acids such as immunostimulatory CpG-DNA, small inhibitory (si-)RNAs, aptamers, carbohydrates, and other biomolecules [vitamin D_3_ or toll-like receptor (TLR) ligands]. Such NP conjugates may mediate (i) efficient delivery, i.e., cellular targeting and uptake, (ii) mucosal adhesion, penetration, and retention, or (iii) immunostimulatory or modulatory effects. Applied in a well-controlled manner, these ligands modify and can thus be used to opimize the safety profile, specificity, and efficacy of a vaccine candidate.

### NPs Mediate Efficient Delivery

Anticytokine therapy has been recognized since the early 2000s (149, 150), and a number of approaches are in the clinic or the pipeline. Examples include antibodies to counteract the effects of TNF-α or IL-1β in inflammatory bowel disease, rheumatoid arthritis, psoriatic arthritis, ankylosing spondylitis, and atherosclerosis. Such antibodies work *via* shifting the immune response from T_H_1 or T_H_17 toward T_H_2 (151, 152). Polyethylene glycol has been regarded as a nanomedical proponent which due to its non-degradable properties under physiological conditions confers a prolonged circulation time of the co-delivered API (153, 154). During AIT, clinical efficacy of a vaccine has to be counterbalanced by a well-defined safety profile of the whole formulation, i.e., API and adjuvant (155). Therefore, the “*hypoallergen concept*” emerged where substances with reduced IgE-binding capacities were used. By genetic engineering or chemical modification (allergoids) the IgE-binding epitopes were disrupted, and hence, higher amounts could be administered at lower risk of side-reactions (156–162).

### NPs Enable Mucosal Adhesion, Tissue Retention, and Penetration

Among the aforementioned ligands, carbohydrates may establish specific as well as non-specific interactions with the human immune system. Therefore, these hydrophilic moieties represent attractive functionalizations for enhanced mucosal delivery *via* the oral, nasal, or dermal routes of application. Upon adhesion with the mucosal or intradermal tissue, prolonged retention may result in a more effective presentation to immunocompetent cells in the dedicated lymphoid tissues (163, 164). Using NPs as a platform for additionally introducing mucoadhesive ligands can improve sublingual AIT, which have been shown effective in ovalbumin-sensitized mouse models (165–169). Table 1 provides a list of potential candidate approaches based on specific (upon binding to lectins) and non-specific (upon hydrophilic interactions of chitosan with mucins) carbohydrate recognition aiming at enhanced efficacy of AIT.

### NPs for Immunostimulation and Modulation toward T_H_1

The response of the immune system against internal or external stimuli can be categorized into two reactions, stimulation or suppression (170). It is possible to push the response either to stimulation or suppression, and this regulation can be used in therapeutic treatment (171, 172). An immune stimulation may be desired for increasing vaccination or cancer treatment efficacy. On the contrary, undesired effects of immune stimulation can result from interactions of leukocytes with NPs (173–175). These may include IFN response, lymphocyte activation, and cytokine storm, leading to severe off-target effects limiting the therapeutic efficacy. Immunosuppression, as observed for inhaled MWCNTs in a mouse model (113), is desired for treatment of hypersensitivities like allergies or autoimmune diseases or in the context of organ transplantation for preventing organ rejection (172, 176, 177). The downside of suppression is that it may lead to an attenuated defense state of the body facilitating infections and cancerous diseases.

The interactions with NPs do not only lead to stimulation or suppression of the immune response but also influence the type of immune response. Both the ability to deviate an immune response from a type 2 to a type 1 response as well as a bias for different types of responses have been described for NPs. Reeducation of tumor-associated macrophages from M2 toward M1 phenotype by NP-mediated induction of pro-inflammatory responses was found effective using the FDA-approved iron oxide NP compound ferumoxytol (58). Similar effects were observed with rabies virus glycoprotein peptide-loaded paclitaxel-carrying biodegradable poly-d,l-lactic-co-glycolic acid (PLGA) NPs in a mouse glioma model, and notably, even crossing of the blood–brain barrier was achieved (66, 178). These polarizing effects may be due to an uptake preference reported for type 2 compared to type 1 macrophages (179). A modulation in immune response was observed using PLGA NPs which were able to downregulate an ongoing T_H_2 response in an allergic BALB/c mouse model (68). Additionally, PLGA NPs have been used to induce a T_H_1 response when delivering the T_H_2-biased peptide hepatitis B surface antigen (180). A potential therapeutic use for PLGA NPs coated with CpG-DNA (TLR9 ligand) and peanut extract was demonstrated when peanut-allergic mice treated with the NPs were protected from anaphylaxis upon challenge and lower levels of T_H_2 cytokines were measured compared to untreated mice (67). Other possible candidate ligands acting as danger signals providing immunodeviation into type 1 include lipopolysaccharide, monophosphoryl lipid-A, cholera or *E. coli* toxins, or flagellin (181–187). Table 1 gives an overview on nanomedical immunomodulatory approaches in particular in respect to AIT, which have recently been reviewed elsewhere (188).

## Concluding Remarks

As for other mechanisms of the immune system (inflammation, type 1 response, tolerance), NPs can modulate type 2 responses in different ways. It is a task for the community, working at the border between immunology and nanotechnology, to understand the parameters leading to NP induced up- or downregulation of type 2 responses. Understanding of such concepts could enable the prediction of the outcomes of human exposure to NPs.

## Author Contributions

All four authors were involved in concept drafting, literature screening, design of display items, writing, and editing of the paper.

## Conflict of Interest Statement

The authors declare that the research was conducted in the absence of any commercial or financial relationships that could be construed as a potential conflict of interest. The reviewer, JS, and handling Editor declared their shared affiliation, and the handling editor states that the process nevertheless met the standards of a fair and objective review.
